# Exendin-4 attenuates adverse cardiac remodelling in streptozocin-induced diabetes via specific actions on infiltrating macrophages

**DOI:** 10.1007/s00395-015-0518-1

**Published:** 2015-11-23

**Authors:** Mitchel Tate, Emma Robinson, Brian D. Green, Barbara J. McDermott, David J. Grieve

**Affiliations:** Wellcome-Wolfson Institute for Experimental Medicine, Queen’s University Belfast, 97 Lisburn Road, Belfast, BT9 7AE UK; Institute for Global Food Security, School of Biological Sciences, Belfast, BT9 5HN UK

**Keywords:** Glucagon-like peptide-1, Experimental diabetes, Cardiac remodelling, Extracellular matrix, Inflammation

## Abstract

In addition to its' established metabolic and cardioprotective effects, glucagon-like peptide-1 (GLP-1) reduces post-infarction heart failure via preferential actions on the extracellular matrix (ECM). Here, we investigated whether the GLP-1 mimetic, exendin-4, modulates cardiac remodelling in experimental diabetes by specifically targeting inflammatory/ECM pathways, which are characteristically dysregulated in this setting. Adult mice were subjected to streptozotocin (STZ) diabetes and infused with exendin-4/insulin/saline from 0 to 4 or 4–12 weeks. Exendin-4 and insulin improved metabolic parameters in diabetic mice after 12 weeks, but only exendin-4 reduced cardiac diastolic dysfunction and interstitial fibrosis in parallel with altered ECM gene expression. Whilst myocardial inflammation was not evident at 12 weeks, CD11b-F4/80^++^ macrophage infiltration at 4 weeks was increased and reduced by exendin-4, together with an improved cytokine profile. Notably, media collected from high glucose-treated macrophages induced cardiac fibroblast differentiation, which was prevented by exendin-4, whilst several cytokines/chemokines were differentially expressed/secreted by exendin-4-treated macrophages, some of which were modulated in STZ exendin-4-treated hearts. Our findings suggest that exendin-4 preferentially protects against ECM remodelling and diastolic dysfunction in experimental diabetes via glucose-dependent modulation of paracrine communication between infiltrating macrophages and resident fibroblasts, thereby indicating that cell-specific targeting of GLP-1 signalling may be a viable therapeutic strategy in this setting.

**Electronic supplementary material:**

The online version of this article (doi:10.1007/s00395-015-0518-1) contains supplementary material, which is available to authorized users.

## Introduction

Diabetes is particularly characterised by increased risk of chronic heart failure (CHF), largely secondary to hypertension or ischemia, which carries a particularly poor prognosis, although the disease itself is an independent risk factor for CHF with some patients developing cardiac dysfunction in the absence of such etiological factors [4, 10]. Whilst the existence of a distinct diabetic cardiomyopathy remains controversial, it is widely held that the diabetic heart undergoes characteristic structural changes independent of ischemia, which undoubtedly underlie enhanced susceptibility to stress [36]. Indeed, a prominent feature is marked collagen accumulation linked with extensive extracellular matrix (ECM) changes, which is the most likely factor underlying cardiac stiffness and subclinical diastolic dysfunction occurring in ~60 % of optimally controlled diabetic patients [13, 38]. This is accompanied by marked changes in cardiomyocyte biology and myocardial inflammation, which is a key driver of cardiac remodelling in this setting, particularly in regard to the ECM [8, 16, 46].

Over the last few years, our group and others have focused on the cardiovascular actions of glucagon-like peptide-1 (GLP-1), whose established metabolic effects have been clinically exploited for glycemic control in type 2 diabetes [14, 41]. It is well known that GLP-1 is an important regulator of normal cardiovascular physiology and also exerts beneficial effects in cardiovascular disease both in the presence or absence of diabetes [28, 41]. Indeed, GLP-1 receptor (GLP-1R) agonists and dipeptidyl peptidase-4 (DPP-4) inhibitors, which increase endogenous GLP-1, are widely reported to protect cardiomyocytes from acute ischemia and improve functional recovery after myocardial infarction (MI) [6, 42]. Importantly, in the context of CHF, two studies have now demonstrated that GLP-1 analogues also exert beneficial actions on post-MI survival and cardiac structure/function in rodent models independent of acute infarct-limiting and metabolic effects [12, 23], suggesting that GLP-1 may directly protect against chronic cardiac remodelling. Notably, our group has recently built on these findings to show that the GLP-1 mimetic, exendin-4, significantly reduces progression of post-MI remodelling in normoglycemic mice via specific actions on myocardial inflammation and the ECM [34], which are characteristic features of diabetes [8]. Indeed, further to the seemingly complex pleiotropic actions of GLP-1 signalling and disappointing results of recent DPP-4 inhibitor cardiovascular trials, it is evident that selective targeting of specific remodelling components may be required to realise the clear therapeutic potential of GLP-1 in this setting [41]. The aim of this study was therefore to investigate the likely beneficial effects of GLP-1 in this setting, testing the hypothesis that exendin-4 modulates development and progression of adverse cardiac remodelling in experimental diabetes by specifically targeting inflammatory and ECM components. Notably, we report for the first time that exendin-4 mediates novel cell-specific glucose-dependent actions on paracrine signalling between infiltrating macrophages and resident fibroblasts, which appear to underlie direct protection against the development of cardiac fibrosis and diastolic dysfunction associated with diabetes.

## Methods

### Experimental animals

Male C57BL/6J mice (8–12 weeks; Harlan UK) were used throughout this study and were housed under constant climatic conditions with free access to food and water. All experimental procedures were performed in accordance with the Guidance on the Operation of the Animals (Scientific Procedures) Act, 1986 (UK) and were approved by the Queen’s University Belfast Animal Welfare and Ethical Review Body.

### Experimental model

Mice were randomised into diabetic and non-diabetic groups at 8–12 weeks of age, which received five consecutive daily injections of streptozotocin (STZ, 55 mg/kg i.p. in citrate buffer, pH 4.5; Sigma-Aldrich, Poole, UK) or vehicle, respectively. STZ-injected mice were confirmed as diabetic if blood glucose concentrations >15 mmol/L (Breeze2, Bayer, Newbury, UK) were evident at 2 weeks. Diabetic and non-diabetic mice were randomly assigned for chronic infusion with either the DPP-4 resistant GLP-1 mimetic, exendin-4 (at a concentration employed in previous in vivo studies; 25 nmol/kg/day; GL Biochem, average purity 90 %) [34], or saline control via an osmotic minipump (Model 1004, Alzet, Cupertino, CA, USA) for either 4 or 8 weeks. For the chronic 8-week treatment study, exendin-4 or vehicle control was administered from 4 weeks after STZ induction, when significantly elevated blood glucose levels were evident, prior to terminal assessment at 12 weeks (Online Resource 1). In this study, a comparator group of diabetic mice received twice daily insulin injections (8 mU/g/day i.p.; Humulin^®^, Eli Lilly, Basingstoke, UK); dose determined in pilot study (Online Resource 2) which induced equivalent reductions in blood glucose versus exendin-4 [27]. For the acute treatment study, animals received exendin-4 or vehicle control 1 day prior to STZ induction and thereafter for a period of 4 weeks (Online Resource 3). At the end of each protocol, animals were either terminally anaesthetised (2 % isoflurane/oxygen) and hearts arrested in diastole by injection of 10 % KCl, excised and fixed in 10 % neutral-buffered formalin solution, or sacrificed by sodium pentobarbitone overdose (200 mg/kg i.p.) prior to excision of hearts which were frozen in liquid nitrogen or subjected to flow cytometry.

### Assessment of plasma lipids and HbA1c

Terminal blood samples were collected by cardiac puncture (after a fasting period of 5 h) into heparinised tubes and centrifuged at 10,000*g* for 10 min to obtain separate cell and plasma fractions. Plasma samples were analysed using enzymatic assay kits (Analox Instruments Ltd, London, UK) for cholesterol (GMRD-084 using cholesterol esterase) and triglyceride (GMRD-195 using lipase) which were detected on a GM7 Micro-Stat Analyser (Analox Instruments Ltd). Glycated haemoglobin, HbA1c, was assessed in cell fractions using a commercially available assay kit (Helena Biosciences, Gateshead, UK), measuring absorbance at 415 nm on a microplate reader (Safire, Tecan, Mannedorf, Switzerland) and was expressed as % of total haemoglobin.

### Echocardiography

Mice were anaesthetised with 1.5 % isofluorane/oxygen, placed on a warming pad, and imaged in the supine position using a Vevo770 ultrasound system with high-frequency 45 MHz RMV707B scanhead (VisualSonics, Amsterdam, The Netherlands). M-mode parasternal short-axis scans at papillary muscle level were used to quantify left ventricular (LV) end-diastolic (LVEDD) and end-systolic diameters (LVESD) from which percent fractional shortening was calculated using the equation (LVEDD − LVESD)/LVEDD × 100. Pulse-wave Doppler was used to assess mitral valve flow (*E*/*A* ratio), LV isovolumetric relaxation time and myocardial performance index, as reliable measures of diastolic function.

### Histology and immunohistochemistry

Following excision, hearts were weighed and measurements normalised to tibial length. All histological analyses were performed using paraffin-embedded LV sections (5 μm). Cardiomyocyte cross-sectional area was determined by H&E staining, analysing cells with centrally located nuclei. Cardiac interstitial fibrosis was assessed by picrosirius red staining (0.1 % w/v), excluding coronary vessels and perivascular regions. Data were quantified by digital image analysis (NIS-Elements, Nikon, London, UK) with the observer blinded to sample identity. Immunocytochemistry for CD45 and F4/80 was performed using rat polyclonal (553076, 1:200; BD Bioscience, Oxford, UK) and rat monoclonal (ab6640, 1:200; Abcam, Cambridge, UK) antibodies, respectively, followed by secondary rabbit anti-rat IgG (P0450, 1:100; Dako, Ely, UK) staining, using diaminobenzidine as the chromogen and nuclear counterstaining with haematoxylin.

Pancreases were harvested into 10 % neutral-buffered formalin, paraffin-embedded, and sectioned (5 μm) prior to staining for glucagon (ab92517, 1:500; Abcam) and insulin (C27C9, 1:200; Cell Signaling, Danvers, MA, USA) using rabbit monoclonal antibodies, followed by incubation with donkey anti-rabbit IgG (ab98502, 1:500; Abcam). Sections were imaged on an epifluorescence microscope (Eclipse 80i, Nikon, London, UK) with the FITC antibodies excited at 488 nm and using an emission filter at a wavelength of 530 nm. Data were quantified by blinded digital image analysis (NIS-Elements, Nikon, London, UK) with the observer blinded to sample identity, and were expressed as % β-cell area and islet number/area per mm^2^ pancreas.

### RNA isolation and quantitative RT-PCR analysis

Total RNA was extracted from LV homogenate or cells using TRI reagent (Sigma-Aldrich, Poole, UK) and cDNA was synthesised by reverse transcription (Life Technologies, Paisley, UK). mRNA expression of procollagen IIIαI, MMP-2, MMP-9, TIMP-2, IL-1β, IL-6, IL-10, α-SMA, CTGF, procollagen IαI, TGF-β_1_, IL-1ra, CXCL10, MIP-1α, MIP-1β, MIP-2, TIMP-1, CD11b, CCL2, and bFGF was analysed by real-time reverse transcription-polymerase chain reaction (RT-PCR) using fluorescent SYBR Green (Prism 7300, Life Technologies, Paisley, UK) and β-actin or GAPDH was used for normalisation (whose expression was shown to remain unaltered between experimental groups in both cells and tissues; GeNorm, Primer Design, Southampton, UK) by the comparative Ct method [49]. Primer sequences are shown in Online Resource 4.

### Flow cytometry

Hearts were collected and the LV separated into RPMI-1640 (Sigma-Aldrich, Poole, UK) and minced in the presence of collagenase type II (1 mg/mL; Invitrogen, Paisley, UK) and DNAse (40 ng/mL; Sigma-Aldrich, Poole, UK), prior to addition of EDTA to prevent cell clumping. After red blood cell lysis using ACK buffer, cells were blocked in 1 % FBS before incubation with the following fluorochrome-conjugated antibody cocktails to assess surface inflammatory cell marker expression by flow cytometry: CD45 (30-F11, 1:250), CD4 (RM4-5, 1:200), CD8 (53–6.7, 1:200), MHC class II (AF6-120.1, 1:200), B220 (RA3-6B2, 1:200), CD11b (M1/70, 1:200), CD117 (2B8, 1:100; all BD Biosciences, Oxford, UK) and F4/80 (MHA497PE, 1:200; AbD Serotec, Oxford, UK). Data were collected on a FACS Canto II flow cytometer (BD Biosciences, Oxford, UK) and analysed using FlowJo software (Tree Star Inc, Ashland, USA).

### Murine bone marrow-derived macrophage culture

Murine bone-marrow derived macrophages (BMDM) were isolated from separate cohorts of untreated C57BL/6J mice, as previously described [34]. Briefly, mice were euthanised (sodium pentobarbitone, 200 mg/kg i.p.) and their femurs isolated before bone marrow cells were flushed out with supplemented DMEM (10 % FBS, and 100 U/mL penicillin and 100 mg/mL streptomycin). Cells were centrifuged and resuspended in ACK lysis buffer (to destroy any contaminating red blood cells) prior to centrifugation and resuspension in DMEM supplemented with L929 cell supernatant (1:6) containing macrophage colony-stimulating factor to induce differentiation. Cells were then seeded into a 150 mm tissue culture plate and after 4 days were scraped into DMEM, centrifuged, and seeded 1:3 into tissue culture plates containing L929 supernatant-supplemented DMEM. After 8 days, cells were split into 6-well plates, each containing 1 million cells, prior to incubation in DMEM without L929 supernatant in the presence of normal (5 mmol/L) or high (25 mmol/L) glucose with or without exendin-4 (1 nmol/L) for 72 h. RNA and cDNA were then prepared for mRNA expression analysis, as described previously, whilst the supernatant (conditioned media) was collected, filtered with a 22 μm filter, and stored at −80 °C for subsequent studies or analysis.

### Murine cardiac fibroblast culture

Murine cardiac fibroblasts were also isolated from untreated C57BL/6J mice, as previously described [34]. Briefly, five mice per isolation were euthanised (sodium pentobarbitone, 200 mg/kg i.p.) and heparinised before their hearts were removed and ventricles isolated prior to mincing and mixing with 4 mL Liberase™ solution (Roche, Burgess Hill, UK) at 37 °C for 8 min. The supernatant was then removed and the process was repeated a further three times with the undigested tissue prior to filtration, centrifugation, and resuspension of the pellet in supplemented DMEM (10 % FBS, 20 mmol/L l-glutamine, 100 U/mL penicillin and 100 mg/mL streptomycin). Cells were then transferred to a 1 % gelatin-coated T75 flask with 10 mL DMEM at 37 °C and cultured in a humidified atmosphere of 5 % CO_2_ for approximately 7 days until 90 % confluent, when they were trypsinised, centrifuged, and reseeded at 1:2 for expansion. At passage 2, cardiac fibroblasts were treated with conditioned media harvested from BMDM (as detailed above), with or without TGF-β (5 ng/mL, to induce myofibroblast differentiation), prior to preparation of RNA/cDNA and real-time RT-PCR analysis of mRNA expression, as described previously.

### Proteome array

Cytokine and chemokine protein expression was assessed in BMDM-conditioned media samples pooled from four preparations using a Proteome Profiler™ antibody array (R&D Systems, Abingdon, UK), as per the manufacturer’s instructions.

### Statistical analysis

Data are expressed as mean ± SEM and were analysed by either a one-way or two-way ANOVA followed by a Bonferroni’s multiple comparison test, using GraphPad Prism software (La Jolla, USA). *P* < 0.05 was considered to be statistically significant.

## Results

### Metabolic changes after 12 weeks STZ diabetes

**Fig. 1:**
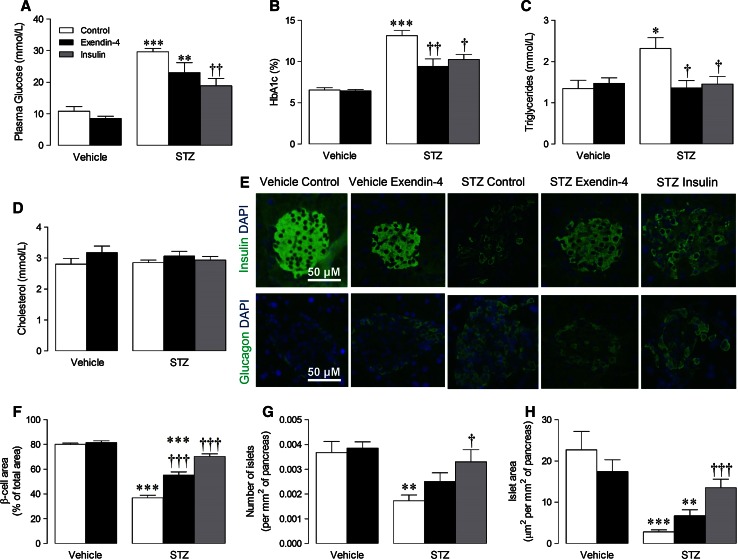
Effect of exendin-4 and insulin on metabolic characteristics after 12 weeks STZ diabetes. Terminal blood assessment of **a** plasma glucose (*n* = 5–18), **b** HbA1c (*n* = 6–13), **c** triglyceride (*n* = 6–14) and **d** cholesterol (*n* = 6–11). **e** Representative images of pancreatic islets stained with insulin and glucagon, with quantification of **f** β-cell area, **g** number of islets and **h** islet area (all *n* = 6–13). *White columns* control; *black columns* exendin-4; *grey columns* insulin; mean ± SEM. *P < 0.05; **P < 0.01; ***P < 0.001, versus corresponding vehicle; ^†^ *P* < 0.05; ^††^ *P* < 0.01; ^†††^ *P* < 0.001 versus STZ control

Serial STZ injections resulted in progressive increases in fasting blood glucose which were maximal by 3 weeks (vehicle control, 9.1 ± 0.5; STZ control, 23.5 ± 1.6 mmol/L; *n* = 6–19, *P* < 0.001) and sustained thereafter. Initially, mice were continuously infused with exendin-4 from 4 to 12 weeks (Online Resource 1), which resulted in significant reduction of blood glucose, HbA1c, and plasma triglycerides versus STZ controls, whilst cholesterol remained unaltered (Fig. 1a–d). To allow separation of indirect and direct actions of exendin-4 on the diabetic heart, an insulin comparator group was included to mimic the observed metabolic effects. Indeed, daily injection of STZ mice with insulin, at a dose determined by a pilot study (Online Resource 2), produced equivalent reductions in blood glucose, HbA1c, and plasma triglycerides versus exendin-4 (Fig. 1a–d). Normal pancreatic morphology, indicated by tightly packed insulin-stained β cells surrounded by a glucagon-positive α-cell “halo” (Fig. 1e), was markedly altered in STZ diabetic mice, reflected by reduced β-cell area and islet number/area with a disordered appearance, effects which were partially rescued by both exendin-4 and insulin (Fig. 1f–h).

### Cardiac function after 12 weeks STZ diabetes

No differences in echocardiographic structure and systolic function were observed between groups, indicated by LV end-systolic/diastolic dimensions and fractional shortening, whilst heart rate also remained unchanged (Table 1). However, diastolic dysfunction evident in STZ diabetic mice was markedly improved by exendin-4, indicated by increased mitral valve *E*/*A* and normalisation of LV isovolumetric relaxation time and myocardial performance index, but notably, these parameters were not altered by insulin.

**Table 1:** Effect of exendin-4 and insulin on cardiac function and morphometry after 12 weeks STZ diabetes

	Vehicle		STZ		
	Control	Exendin-4	Control	Exendin-4	Insulin
*Echocardiography data*					
*n*	6	6	16	10	6
Heart rate (bpm)	434 ± 13	430 ± 31	415 ± 10	427 ± 18	415 ± 8
LVESD (mm)	2.64 ± 0.05	2.44 ± 0.07	2.47 ± 0.06	2.62 ± 0.05	2.64 ± 0.06
LVEDD (mm)	3.77 ± 0.09	4.05 ± 0.04	3.78 ± 0.07	3.91 ± 0.08	3.92 ± 0.07
Fractional shortening (%)	37.0 ± 1.1	32.7 ± 1.0	34.1 ± 0.8	34.4 ± 0.9	35.8 ± 1.2
MV *E*/*A*	2.11 ± 0.05	2.06 ± 0.04	1.21 ± 0.06***	1.58 ± 0.05***^,†††^	1.29 ± 0.08
IVRT (ms)	16.4 ± 1.7	17.9 ± 0.6	21.4 ± 1.3	15.6 ± 1.9^†^	20.3 ± 0.9
MPI	0.51 ± 0.03	0.57 ± 0.04	0.62 ± 0.02*	0.51 ± 0.04^†^	0.62 ± 0.03
*Morphometric data*					
*n*	6	6	19	12	6
Body weight (g)	28.2 ± 1.2	27.9 ± 0.6	25 ± 0.6***	25.3 ± 0.9**	26.5 ± 0.3
Tibial length (mm)	18.5 ± 0.1	19.1 ± 0.2	18.4 ± 0.2	18.6 ± 0.2	18.8 ± 0.2
LV weight (mg)	81.8 ± 5.4	90.2 ± 4.7	74.9 ± 2.3	72.4 ± 2.2**	77.9 ± 4.4
LV weight/tibial length	4.33 ± 0.33	4.71 ± 0.22	3.91 ± 0.13*	3.84 ± 0.11	4.15 ± 0.22

Mean ± SEM

*LVESD* LV end-systolic diameter, *LVEDD* LV end-diastolic diameter, *MV E/A* mitral valve *E*/*A* ratio, *IVRT* isovolumetric relaxation time, *MPI* myocardial performance index

* *P* < 0.05; ** *P* < 0.01; *** P < 0.001 versus corresponding vehicle

^†^ *P* < 0.05; ^†††^ *P* < 0.001 versus STZ control

### Cardiac remodelling

**Fig. 2:**
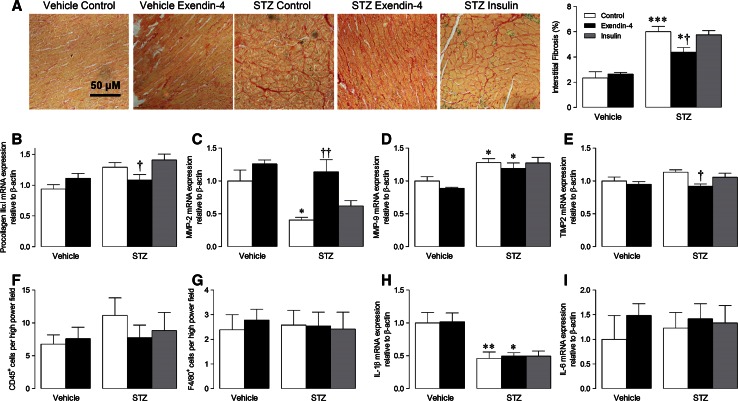
Effect of exendin-4 on ECM remodelling and myocardial inflammation after 12 weeks STZ diabetes. **a** Representative LV sections stained with picrosirius red to assess interstitial fibrosis and quantification data (*n* = 6–13). **b**–**e**, **h**, **i** mRNA expression of ECM genes/cytokines in LV tissue (*n* = 5–8). **f**–**g** Quantification of LV sections probed for CD45 and F4/80 to assess leukocyte and macrophage infiltration, respectively (*n* = 4–8). *White columns* control; *black columns* exendin-4; *grey columns* insulin; mean ± SEM. *P < 0.05; **P < 0.01; ***P < 0.001 versus corresponding vehicle; ^†^ *P* < 0.05; ^††^ *P* < 0.01 versus corresponding control

Neither exendin-4 nor insulin had any effect on body weight, cardiac morphometrics or cardiomyocyte cross-sectional area in STZ mice after 12 weeks (Table 1; Online Resource 5). However, cardiac interstitial fibrosis was markedly increased in STZ control hearts, which, consistent with the diastolic function data, was attenuated by exendin-4, but not insulin (Fig. 2a). Similarly, mRNA expression of several important ECM genes (procollagen IIIαI, MMP-2, and TIMP2) was significantly modulated in STZ mice by exendin-4-but not insulin (Fig. 2b–e).

### Myocardial inflammation

**Fig. 3:**
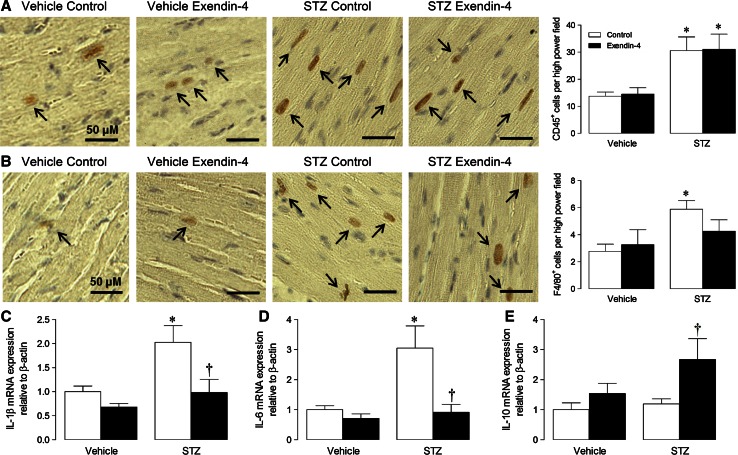
Effect of exendin-4 on myocardial inflammation after 4 weeks STZ diabetes. Representative LV sections probed for **a** CD45 and **b** F4/80, to assess leukocyte and macrophage infiltration, respectively, together with mean quantification data analysing ten high power fields per section (*n* = 5–6). **c**–**e** cytokine mRNA expression in LV tissue (*n* = 5–7). *White columns* control; *black columns* exendin-4; mean ± SEM. *P < 0.05; ***P < 0.001 versus corresponding vehicle; ^†^ *P* < 0.05 versus STZ control

Although exendin-4 markedly improved STZ-induced ECM remodelling and diastolic dysfunction, which may be largely driven by inflammation [36], myocardial infiltration of CD45^+^ leukocytes and F4/80^+^ macrophages were not significantly different between groups after 12 weeks (Fig. 2f, g), whilst no effect of exendin-4 on pro-inflammatory cytokines was observed (Fig. 2h, i). A pilot study was subsequently performed to characterise the time course of STZ-induced inflammation by flow cytometry (Online Resource 6). Notably, significant myocardial inflammatory cell infiltration was evident at 4 weeks (but not 8 or 12 weeks), indicated by increased CD11b-F4/80^++^ macrophages and CD117^+^ mast cells, with the former contributing ~5 times more cells (although total CD45^+^ cells were unaltered). As it appeared that acute myocardial inflammation is particularly important in STZ diabetes, which may drive subsequent fibrosis and diastolic dysfunction, a short-term model was employed in mice which were infused with exendin-4 1 day prior to STZ induction and for 4 weeks (Online Resource 3). Indeed, myocardial infiltration of CD11b-F4/80^++^ macrophages (but not CD45^+^ leukocytes; analysed using ten high power fields per section) was significantly increased in STZ hearts after 4 weeks, and clearly reduced by exendin-4, although numbers of CD3–CD4–CD8^+++^ T cells, B220^+^ B cells, and MHC class II^+^ antigen-presenting cells remained similar between groups (Table 2). Whilst further immunohistochemical analysis demonstrated marked CD45^+^ leukocyte infiltration in STZ mice which was unaltered by exendin-4 (Fig. 3a), significantly increased F4/80^+^ macrophages in diabetic hearts were reduced in exendin-4-treated mice (Fig. 3b). Similarly, increased pro-inflammatory IL-1β and IL-6 expression in STZ hearts was decreased by exendin-4, whilst anti-inflammatory IL-10 expression was induced by exendin-4 (Fig. 3c–e). Indeed, consistent with the 12-week model, diabetic mice treated with exendin-4 for 4 weeks coincident with STZ induction demonstrated preserved diastolic dysfunction, indicated by elevated mitral valve *E*/*A* (Online Resource 7), suggesting that the cardioprotective actions of exendin-4 may occur via modulation of acute inflammation.

**Table 2:** Flow cytometry characterisation of cardiac inflammatory cells after 4 weeks STZ diabetes

	Vehicle		STZ	
	Control	Exendin-4	Control	Exendin-4
CD45^+^/100,000 total cells	105 ± 10	139 ± 21	174 ± 18	205 ± 25
CD11b-F4/80^++^/CD45^+^ cells (%)	13.7 ± 0.9	12.2 ± 0.7	20.6 ± 1.4***	15.4 ± 1.1^†^
CD4^+^/CD45^+^ cells (%)	6.6 ± 0.9	8.1 ± 0.7	7.0 ± 0.3	7.1 ± 1.1
CD8^+^/CD45^+^ cells (%)	5.4 ± 0.3	6.5 ± 0.3	5.7 ± 0.3	8.3 ± 1.8
MHCII^+^/CD45^+^ cells (%)	58.9 ± 2.4	52.2 ± 2.4	55.9 ± 2.2	54.1 ± 1.2
B220^+^/CD45^+^ cells (%)	48.6 ± 4.1	44.7 ± 1.4	47.1 ± 3.2	47.8 ± 2.3

Each value represents relative frequency versus total CD45^+^ cells. Mean ± SEM (*n* = 6–8)

*** *P* < 0.001 versus corresponding vehicle

^†^ *P* < 0.05 versus STZ control

### Isolated cell studies

**Fig. 4:**
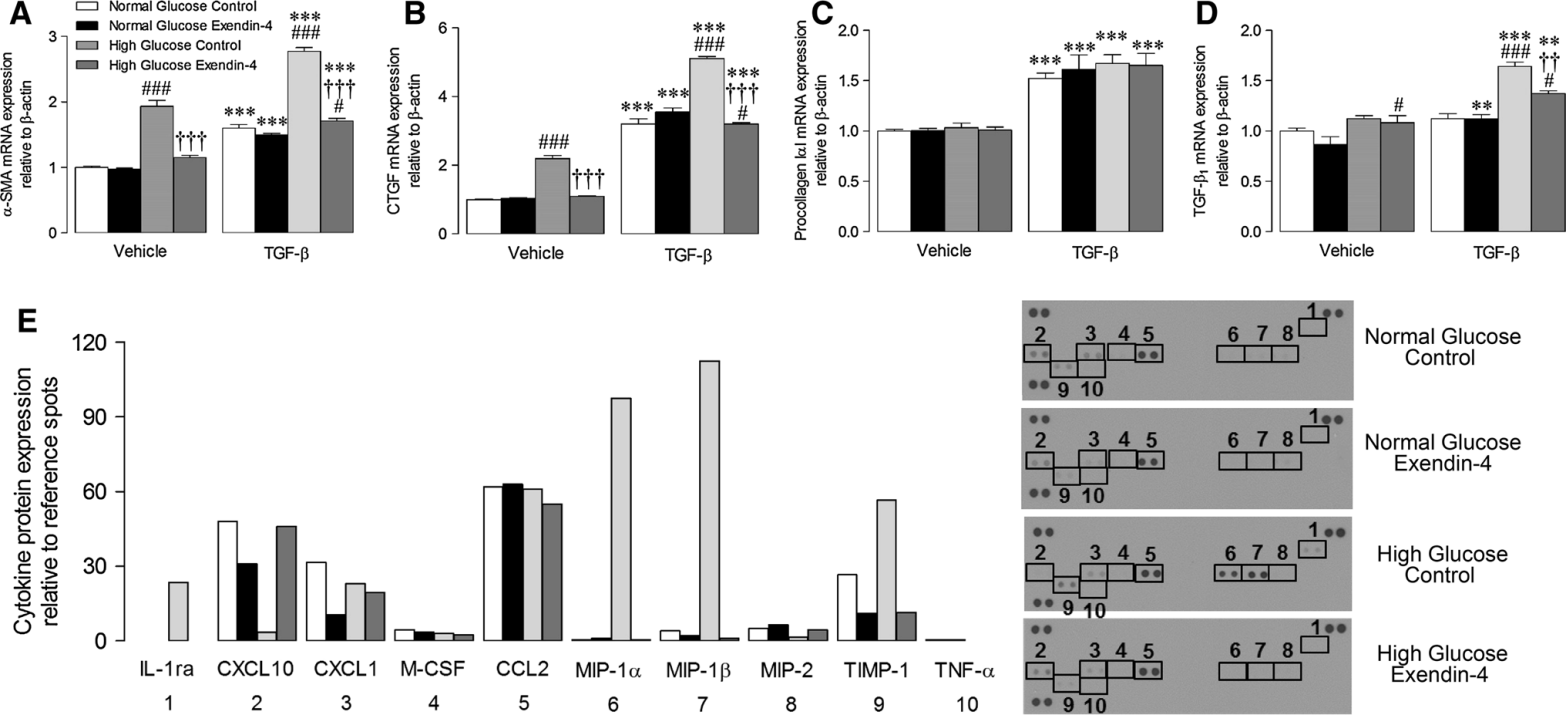
Effect of exendin-4 on paracrine communication between bone marrow-derived macrophages and cardiac fibroblasts. **a**–**d** mRNA expression of myofibroblast differentiation markers in basal and TGF-β-stimulated cardiac fibroblasts incubated in conditioned media from BMDM treated with normal/high glucose with/without exendin-4 for 24 h (*n* = 6). **e** Cytokine/chemokine array blots from BMDM-conditioned media with quantification of protein expression (pooled from four preparations). *White columns* normal glucose control; *black columns* normal glucose exendin-4; *light grey columns* high glucose control; *dark grey columns* high glucose exendin-4; mean ± SEM. **P < 0.01; ***P < 0.001 versus corresponding vehicle; ^††^ *P* < 0.01; ^†††^ *P* < 0.001 versus corresponding glucose control; ^#^ *P* < 0.05; ^###^ *P* < 0.001 versus corresponding normal glucose

**Fig. 5:**
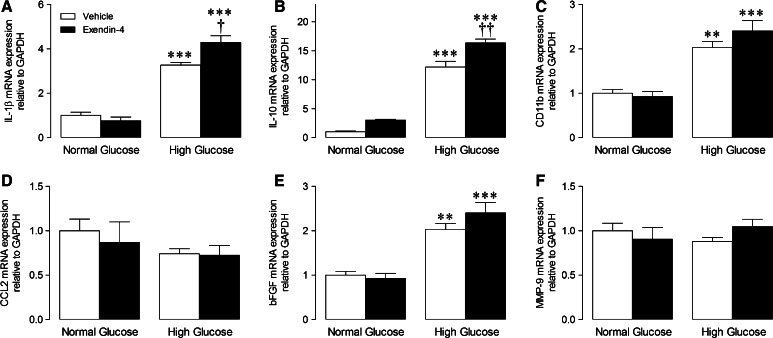
Effect of exendin-4 on mRNA expression in bone marrow derived macrophages incubated in normal and high glucose for 72 h. Real-time RT-PCR analysis of transcript levels of **a** IL-1β, **b** IL-10, **c** CD11b, **d** CCL2, **e** bFGF, and **f** MMP-9 were analysed (*n* = 4). *White columns* control; *black columns* exendin-4; mean ± SEM. **P < 0.01; ***P < 0.001 versus corresponding vehicle, ^†^ *P* < 0.05; ^††^ *P* < 0.01, versus normal glucose exendin-4

**Fig. 6:**
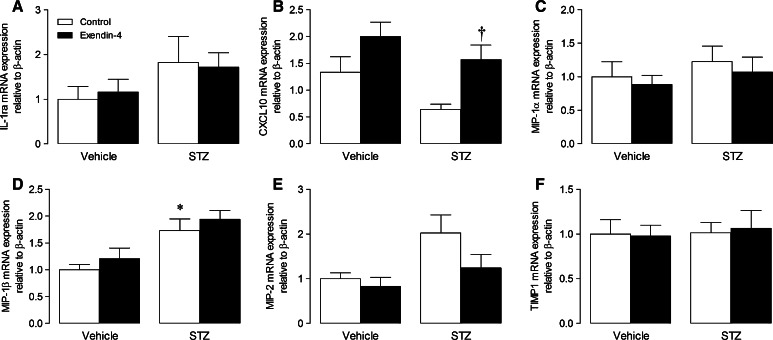
Effect of exendin-4 on cardiac candidate cytokine and chemokine expression after 4 weeks STZ diabetes. **a**–**f** Cytokine and chemokine mRNA expression in LV tissue (*n* = 5–7). *White columns* control; *black columns* exendin-4; mean ± SEM. *P < 0.05 versus vehicle control; ^†^ *P* < 0.05 versus STZ control

Our previous study indicated that exendin-4 does not directly influence murine cardiac fibroblasts, but may rather modulate the macrophage secretome [34], highlighting paracrine communication as a likely mechanism underlying improved ECM remodelling and diastolic function in exendin-4-treated STZ mice. We therefore performed a series of studies to investigate effects of exendin-4 on BMDM mRNA expression, secretion, and paracrine effects on cardiac fibroblasts under both normal and high glucose conditions. Interestingly, conditioned media from BMDM maintained in high glucose for 72 h induced myofibroblast differentiation in unstimulated cells, indicated by increased expression of α-SMA and CTGF, an effect not seen in normoglycemia but which was accentuated in the presence of TGF-β, evident by further induction of α-SMA and CTGF together with TGF-β_1_ (Fig. 4a–d). Notably, these changes were virtually abolished in fibroblasts incubated in conditioned media from exendin-4-treated BMDM. To identify potential paracrine mediators, BMDM-conditioned media pooled from each experimental group was analysed by proteome array, which identified several cytokines/chemokines differentially secreted by exendin-4-treated cells in high glucose (Fig. 4e). Intracellular mRNA expression of key cytokines was also induced in high glucose-treated BMDM (IL-1β, IL-10, CD11b, and bFGF) and further increased by exendin-4 (Fig. 5). Notably, mRNA expression of one of the candidate BMDM-secreted cytokines/chemokines identified by the proteome array, CXCL10, was significantly modulated in LV tissue from STZ exendin-4-treated mice at 4 weeks (Fig. 6b), together with IL-1β and IL-10 (Fig. 3c, e, which were also induced by high glucose; Fig. 5a, b), highlighting their potential importance in vivo, whilst others tended towards change (e.g. MIP-2; Fig. 6e).

## Discussion

This is the first study to report specific effects of the GLP-1 mimetic, exendin-4, on adverse cardiac remodelling in experimental diabetes, which occur at least partly independently of its established glucose-lowering actions. Exendin-4 preferentially reduced ECM remodelling and diastolic dysfunction, which was associated with specific attenuation of acute myocardial macrophage infiltration and altered inflammatory gene expression. Notably, complementary cell studies revealed direct anti-inflammatory effects of exendin-4 on BMDM under both normoglycemic and hyperglycemic conditions, and highlighted novel glucose-dependent paracrine inhibition of cardiac fibroblast differentiation, suggesting that the cardioprotective actions of exendin-4 in diabetes may occur via specific modulation of infiltrating macrophages.

The rationale for this study was based on recent reports that GLP-1 analogues improve post-MI remodelling in rodent models and our own work highlighting specific modulation of ECM and inflammatory components [12, 23, 34], which are characteristic features of the diabetic heart. Indeed, this supposition is supported by reports that cardiac GLP-1R expression may be less ubiquitous than previously thought and the persistence of liraglutide-induced cardioprotection in mice with cardiomyocyte-specific GLP-1R deletion [41, 44], suggesting that the cardiac actions of GLP-1 may be mediated by an alternative cell type. To address this key question, we chose to employ an established murine model of experimental STZ diabetes using a combined in vivo/ex vivo/in vitro approach. Whilst a type 2 model may be more clinically relevant, it should be noted that, of these, only db/db mice show discernible cardiac dysfunction after a prolonged period whilst data analysis in such models may be confused by the cardiovascular effects of obesity and/or dysfunctional leptin signalling, so STZ diabetes (which shares common features with type 2 diabetes) was considered to be the most appropriate choice for our study [1, 3, 5, 37]. Importantly, the employed low-dose STZ induction protocol promotes an immune β-cell response causing progressive β-cell damage, leading to local inflammation and insulitis as seen in clinical diabetes [7], thereby avoiding potentially confounding STZ toxicity associated with other models. STZ diabetes is also associated with significant cardiac remodelling at 12 weeks, by which time diastolic dysfunction and fibrosis are clearly evident, which are characteristic of both type 1 and type 2 diabetes, and this model has been widely employed for such studies [18, 20, 46]. Notably, exendin-4 was infused from 4 weeks, at a concentration comparable to previous experimental studies [32, 34], once elevated glucose levels had been achieved, to further reflect the likely clinical scenario.

The initial clinical manifestation of cardiac dysfunction in diabetes is often asymptomatic and only emerges once patients present with ischemic or hypertensive CHF [36]. Despite significant advances in elucidation of the underlying pathogenesis, separation of the undoubted direct cardiac effects of diabetes and the indirect contribution of associated cardiovascular risk factors has remained difficult, which is critical as 60 % of optimally managed diabetic patients have diastolic dysfunction [13, 30]. Indeed, GLP-1 exerts numerous extra-pancreatic actions, under both physiological and pathophysiological conditions, including reduction of diabetic and cardiovascular risk factors [14, 41]. However, both GLP-1 analogues and DPP-4 inhibitors confer protection against cardiac dysfunction/remodelling associated with experimental MI, dilated cardiomyopathy, and hypertensive CHF in the absence of metabolic changes [15, 23, 31, 33], indicating that GLP-1 exerts direct cardiac effects. To dissect such actions of exendin-4 in experimental diabetes, we performed comprehensive metabolic assessment of our animals and also included an insulin comparator group. Blood glucose, HbA1c, and triglycerides were elevated in STZ animals and reduced to comparable levels by exendin-4 and insulin. Pancreatic morphology was also obviously impaired by STZ (although the low-dose model does not completely ablate β cells), and both exendin-4 and insulin promoted partial regeneration, which is consistent with previous reports and may occur due to mobilisation of progenitor cell populations [7, 11, 48]. Notably, exendin-4 markedly improved both diastolic function (as indicated by mitral valve *E*/*A*, LV isovolumetric relaxation time, and myocardial performance index) and ECM remodelling in STZ mice, whereas insulin-treated animals, which demonstrated similar metabolic benefits (and indeed tended to exhibit lower blood glucose and better pancreatic morphology), displayed equivalent cardiac dysfunction/remodelling to STZ controls, strongly suggesting that exendin-4 exerts direct cardioprotective actions in diabetes.

Consistent with our previous study, exendin-4 (and insulin) had no effect on cardiac morphometry or cardiomyocyte area in diabetic animals, although both body weight and LV mass were reduced by STZ, which are recognised features of this model [34, 45]. Since ECM remodelling and fibrosis are the natural consequence of inflammation, which is particularly evident in diabetes [8, 46], we primarily focused on characterising effects of exendin-4 on the myocardial inflammatory response in STZ mice. As cardiac inflammation had resolved by 12 weeks, and subsequent flow cytometry analysis demonstrated early and specific infiltration of CD11b-F4/80^++^ macrophages and CD117^+^ mast cells in diabetic hearts, further studies were conducted in which exendin-4 was infused concomitant with STZ induction to investigate acute inflammatory effects. In this case, exendin-4 induced marked attenuation of macrophage infiltration, whilst mast cells were unaffected, suggesting that the observed effects on myocardial function/remodelling in the 12-week model may occur secondary to specific modulation of acute macrophage function (although the source of these cells cannot be concluded from our data). Indeed, macrophage infiltration is reported to be increased in diabetic hearts, where these cells display a switch from a M2 anti-inflammatory towards a M1 pro-inflammatory profile, together with elevated cytokine levels [35, 43]. Notably, in addition to our previous work highlighting macrophage-specific effects of exendin-4 post-MI [34], many basic and clinical studies are broadly supportive of persistent anti-inflammatory actions of GLP-1, including specific reduction of TNF-α, IL-6, TLR-2/4, NF-κβ, and MMP-9, which are key drivers of cardiac remodelling [9, 17, 40]. Furthermore, several studies report specific actions of GLP-1 on macrophage function, supporting our suggestion that this represents the key inflammatory cell type underlying its cardioprotective effects. For example, exendin-4 inhibits monocyte adhesion in apoE^−/−^ mice and suppresses LPS-induced macrophage expression of pro-inflammatory cytokines, whilst the DPP-4 inhibitor, sitagliptin, exerts similar cAMP-dependent anti-inflammatory actions in human macrophages and reduces macrophage-mediated vascular inflammation in angiotensin II-infused apoE^−/−^ mice [2, 24, 26]. Of particular relevance to our study, a GLP-1-producing recombinant adenovirus inhibits adipose tissue macrophage infiltration and inflammation in ob/ob diabetic mice [22]. Importantly, comparable effects have been reported clinically, with the GLP-1R agonist, liraglutide, decreasing circulating levels of the inflammatory macrophage activation molecule, sCD163, in diabetic patients [17].

It is becoming increasingly evident that inflammation plays a key role in the pathogenesis of cardiac remodelling, particularly in diabetes, and that macrophages may be critically involved. This putative role is supported by the heterogeneous nature of monocyte/macrophage populations, which exhibit remarkable plasticity, allowing them to alter physiological function in response to their microenvironment [29]. Notably, macrophages are involved throughout the fibrotic response, during initiation, progression, and resolution, and it appears that distinct macrophage populations exert both pro-fibrotic and anti-fibrotic actions [47]. Indeed, stimulated macrophages induce IL-6 in cardiac fibroblasts and subsequent production of TGFβ_1_ and Smad3 phosphorylation, thereby promoting myofibroblast differentiation both in vivo and in vitro [25], providing support for macrophage–fibroblast communication as an important driver of cardiac remodelling. With respect to our study, it is conceivable that specific modulation of macrophage function by GLP-1 may underlie many of its reported cardioprotective actions due to indirect effects on the two primary effector cell types, ventricular cardiomyocytes and fibroblasts, which do not appear to express the GLP-1R, which is abundantly localised in monocytes and macrophages including mouse BMDM employed in this study [34, 39, 44].

In this regard, we have previously reported that exendin-4 has no effect on cardiac myofibroblast differentiation in normoglycemia, but does modulate basal macrophage inflammatory gene expression and secretion [34]. Here, we extended these findings to demonstrate that conditioned media harvested from BMDM maintained in high glucose was able to induce myofibroblast differentiation, which was inhibited by exendin-4, highlighting paracrine communication between these cells as a potential target of GLP-1. Notably, the concentration of exendin-4 used in these studies (1 nmol/L) is equivalent to circulating levels typically found in diabetic patients receiving exenatide [21]. Interestingly, the inhibitory effect of exendin-4 on myofibroblast differentiation was not observed in normoglycemia, which is consistent with a previous report that the ex vivo infarct-reducing actions of the DPP-4 inhibitors, sitagliptin and vildagliptin, are only evident at elevated glucose concentrations (>7 mmol/L), with similar effects observed in vivo in diabetic but not normoglycemic rats [15], suggesting that the cardioprotective actions of GLP-1 may be glucose-dependent. Although in vitro experimental models fail to mimic the complex interactions that regulate cell signalling in vivo, the findings of our complementary cell studies appear to corroborate interpretation of our in vivo data in suggesting that exendin-4 inhibits macrophage recruitment to the diabetic heart and modulates subsequent paracrine signalling, thereby reducing myofibroblast differentiation and resultant ECM remodelling and diastolic dysfunction. Importantly, of the cytokines/chemokines that were differentially expressed/secreted by high glucose-treated BMDM in response to exendin-4, several were also altered in cardiac tissue from diabetic mice (e.g. CXCL10, IL-1β, and IL-10), indicating that they may play a role in regulating ECM remodelling. However, it should be noted that the in vivo myocardial inflammatory response in diabetes is highly complex and temporal in nature, so it is possible that some of the identified mediators may not be relevant to the cardioprotective effects of exendin-4, whilst others may have been overlooked. Nonetheless, these data are clearly exciting in highlighting potential cell-specific effects of exendin-4 which may underlie reduced cardiac remodelling in experimental diabetes, and thereby warrant detailed further investigation.

Taken together, our data indicate that exendin-4 specifically targets macrophage infiltration and function to reduce adverse cardiac remodelling associated with experimental diabetes. Whilst CHF in diabetic patients is an undoubted multifactorial process, which is commonly associated with ischemia and hypertension, it is clear that the diabetic milieu is directly damaging to the heart and thereby makes a significant contribution to disease progression. In this regard, accumulating evidence implicates aberrant inflammation as a key driver of pathogenic cardiac remodelling, particularly in diabetes [19], so it is highly conceivable that inflammatory pathways, such as those identified in this study, whose activation precedes the development of contractile dysfunction and fibrosis, may represent potential therapeutic targets. Although further work is clearly required to delineate underlying mechanisms, our findings are clearly exciting and suggest that selective cell-specific targeting of GLP-1 signalling may represent a novel approach for the prevention/treatment of CHF which is a major complication of both type 1 and type 2 diabetes.

## Electronic supplementary material

Supplementary material 1 (DOCX 1058 kb)
