# Supplementary material for: Exendin-4 attenuates adverse cardiac remodelling in streptozocin-induced diabetes via specific actions on infiltrating macrophages
Source: Basic Res Cardiol. 2015 Nov 23;111:1. doi: 10.1007/s00395-015-0518-1 (PMC4656717; doi:10.1007/s00395-015-0518-1)
Supplement: Supplementary file 1 — Supplementary material 1 (DOCX 1058 kb) [file 395_2015_518_MOESM1_ESM.docx]

**SUPPORTING INFORMATION**

**Exendin-4 attenuates ADVERSE CARDIAC REMODELLING IN STREPTOZOCIN-INDUCED DIABETES via SPECIFIC actions on infILTRATING MACROPHAGES**

Mitchel Tate^1^, Emma Robinson^1^, Brian D. Green^2^, Barbara J. McDermott^1^, David J. Grieve^1^

Queen’s University Belfast, ^1^Wellcome-Wolfson Institute for Experimental Medicine, Belfast BT9 7AE, UK and ^2^Institute for Global Food Security, School of Biological Sciences, Belfast BT9 5HN, UK

**ONLINE RESOURCE 1**


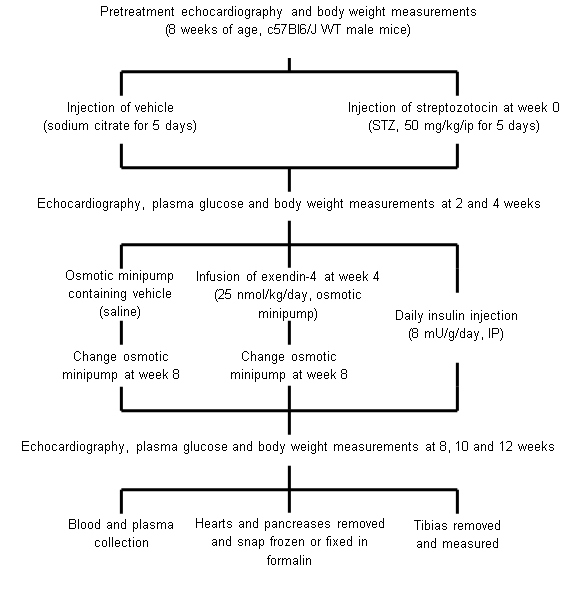


Schematic representation of the 12 week study plan. Mice were injected with STZ or vehicle 4 weeks prior to treatment with either exendin-4, insulin or saline. At 12 weeks, animals were sacrificed and tissue collected for further analyses. Echocardiographic assessment of cardiac function was performed at regular intervals throughout the study.

**ONLINE RESOURCE 2**


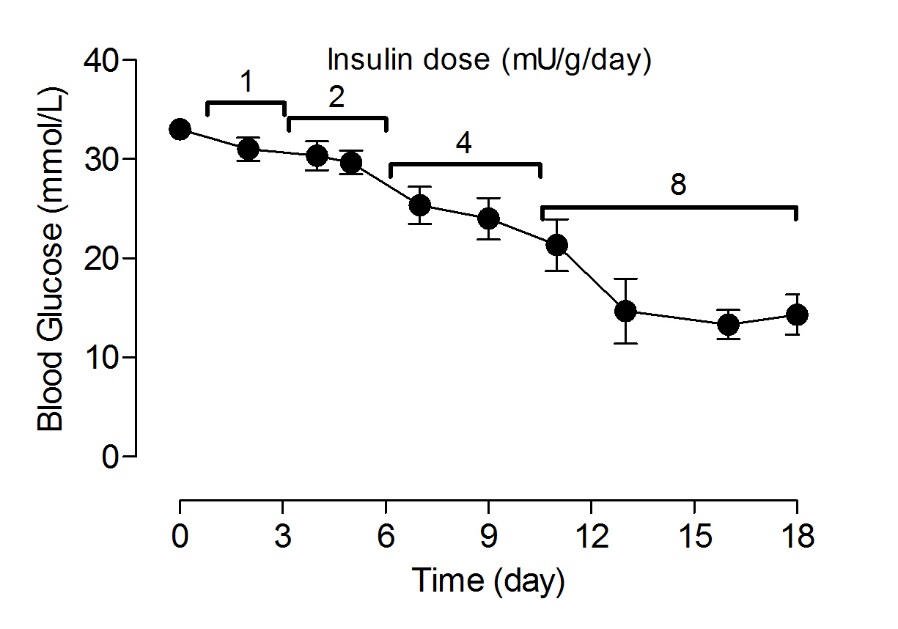


Pilot study conducted in STZ-treated mice from 4 weeks to determine the dose of insulin that would induce equivalent reductions in blood glucose compared to exendin-4. Mice (*n*=3) were injected twice daily with insulin at increasing concentrations, based on a previous study [27], and effects on blood glucose levels were serially assessed.

**ONLINE RESOURCE 3**


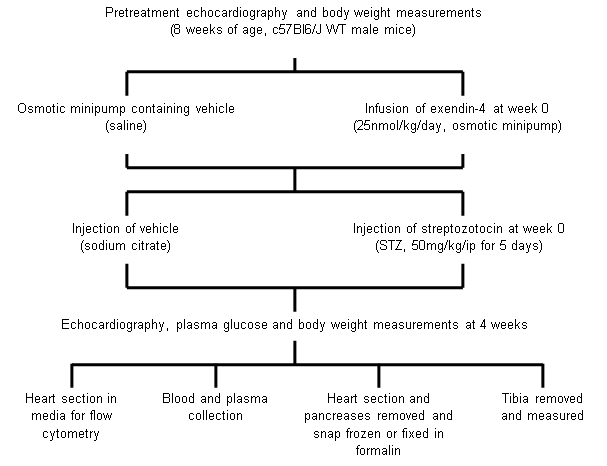


Schematic representation of the 4 week study plan. Mice were continuously infused with exendin-4 or saline one day prior to injection with STZ or vehicle. At 4 weeks, animals were sacrificed and tissue collected for further analyses. Echocardiographic assessment of cardiac function was performed at baseline and prior to termination.

**ONLINE RESOURCE 4:** Real-time RT-PCR primer sequences

| **Gene** | **Forward Primer (5’-3’)** | **Reverse Primer (5’-3’)** |
| --- | --- | --- |
| **Procollagen IIIαI** | AGGAGCCAGTGGCCATAATG | TGACCATCTGATCCAGGGTTTC |
| **MMP-2** | GACAAGTTCTGGAGATACAATGAAGTG | CAGGTTATCAGGGATGGCATTC |
| **MMP-9** | GTGATCCCCACTTACTATGGAAACTC | GTGCTACACCAAGGCGTGC |
| **TIMP-2** | GATTCAGTATGAGATCAAGCAGATAAAGA | GCGAGACCCCGCACACT |
| **IL-1β** | TGTGGCTGTGGAGAAGCTGT | CAGCTCATATGGGTCCGAGA |
| **IL-6** | CACGGCCTTCCCTACTTCAC | TGCAAGTGCATCATCGTTGT |
| **IL-10** | TGCAGGACTTTAAGGGTTACTTGG | GGCCTTGTAGACACCTTGGTC |
| **α-SMA** | GCAGCCCAGCCAAGCACTGTCAGGAAT | AGCCCAGAGCCATTGTCACACACCAAGG |
| **CTGF** | GCTGCCTACCGACTGGAAGAC | GAACAGGCGCTCCACTCTG |
| **Procollagen IαI** | CCTCAGGGTATTGCTGGACAAC | TTGATCCAGAAGGACCTTGTTTG |
| **TGF-β_1_** | GCTGCTGACCCCCACTGATA | AAGCCCTGTATTCCGTCTCCTT |
| **IL-1ra** | TTGCTGTGGCCTCGGGATGG | GTTTGATATTTGGTCCTTGTAAG |
| **CXCL10** | GACGGTCCGCTGCAACTG | GCTTCCCTATGGCCCTCATT |
| **MIP-1α** | ATGAAGGTCTCCACCACTG | GCATTCAGTTCCAGGTCA |
| **MIP-1β** | CCATGAAGCTCTGCGTGTCTG | GGCTTGGAGCAAAGACTGCTG |
| **MIP-2** | CGCTGTCAATGCCTGAAGAC | ACACTCAAGCTCTGGATGTTCTTG |
| **TIMP1** | ACTCGGACCTGGTCATAAGGGC | TTCCGTGGCAGGCAAGCAAAGT |
| **CD11b** | AAACCACAGTCCCGCAGAGA | CGTGTTCACCAGCTGGCTTA |
| **CCL2** | TGTAGTTTTTGTCACCAAGCTCAAG | GTAGGTTCTGATCTCATTTGGTTCC |
| **bFGF** | AAGAGCGACCCACACGTCAAAC | GTAACACACTTAGAAGCCAGCAGCC |
| **CCL2** | TGTAGTTTTTGTCACCAAGCTCAAG | GTAGGTTCTGATCTCATTTGGTTCC |
| **β-Actin** | CGTGAAAAGATGACCCAGATCA | TGGTACGACCAGAGGCATACAG |
| **GAPDH** | ACTTTGTCAAGCTCATTTCC | GCAGCGAACTTTATTGATG |

**ONLINE RESOURCE 5**


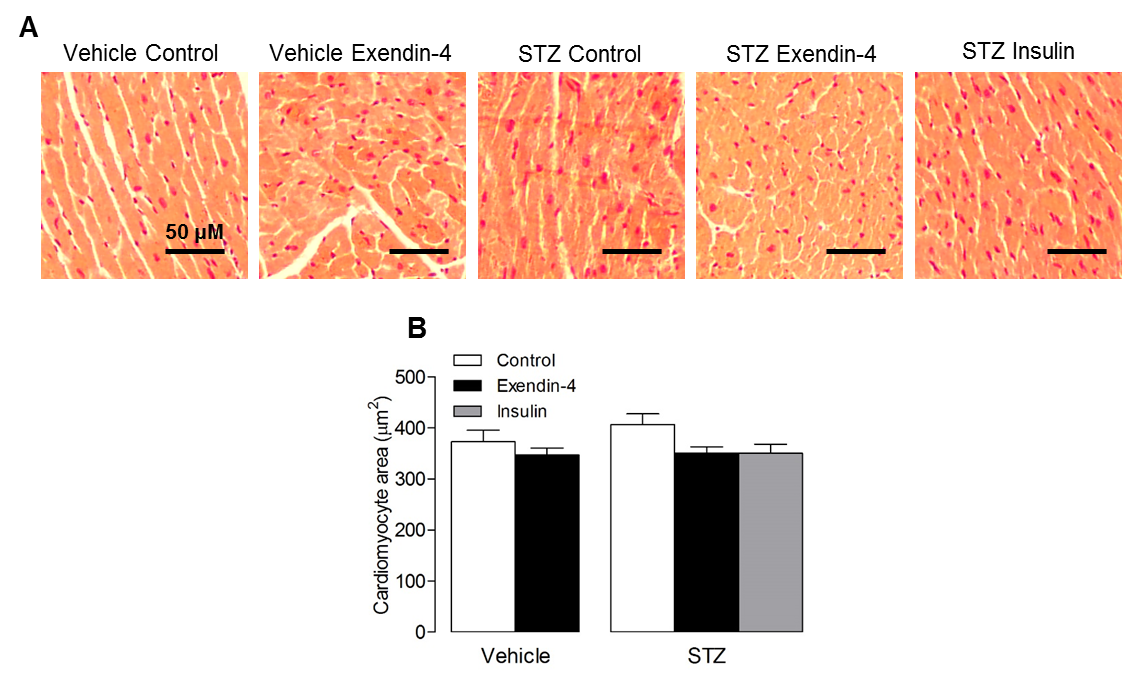


Effect of exendin-4 and insulin on cardiomyocyte remodelling in a 12-week model of STZ diabetes. (A) Representative examples of LV sections stained with H&E to delineate cardiomyocytes, (B) cardiomyocyte cross-sectional area. White columns, control; black columns, exendin-4; grey columns, insulin; mean ± SEM (*n*=6-13).


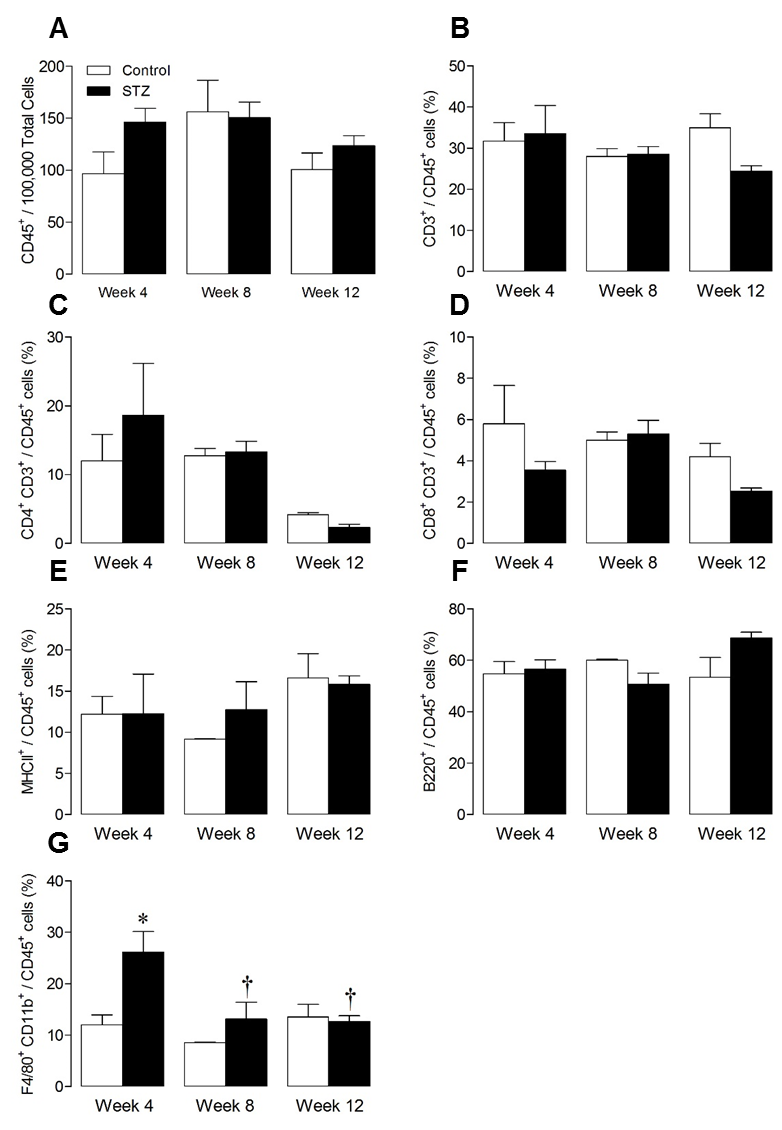
 **ONLINE RESOURCE 6**

Profiling of cardiac inflammatory cells during progression of STZ diabetes by flow cytometry. (A) Total CD45^+^ cells per 100,000 cells was analysed before relative frequencies of (B) CD3^+^, (C) CD3-CD4^++^, (D) CD3-CD8^++^, (E) MHCII^+^, (F) B220^+^, (G) CD11b-F4/80^++^ cells were assessed. White columns, control; black columns, STZ; mean ± SEM (*n*=4). *P<0.05 versus 4 week control, ^†^P<0.05 versus 4 week STZ.

**ONLINE RESOURCE 7**

**
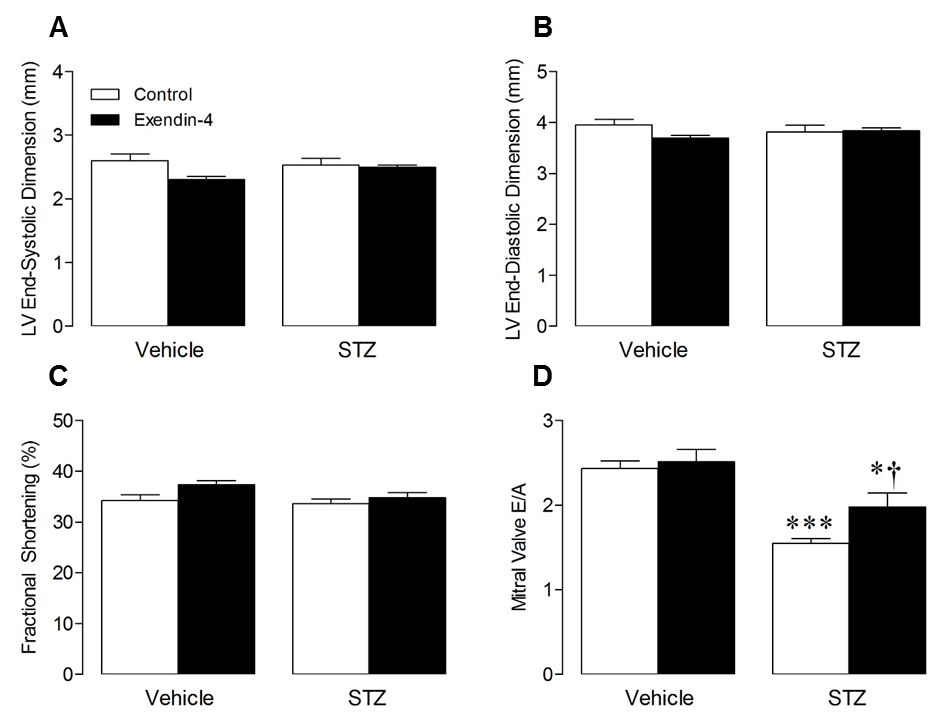
**

Effect of exendin-4 on cardiac function in a 4-week model of STZ diabetes. (A) LV end-systolic dimension, (B) LV end-diastolic dimension, (C) fractional shortening and (D) mitral valve E/A ratio, as assessed by echocardiography. White columns, control; black columns, exendin-4; mean±SEM (*n*=5-6). ^*^P<0.05, ^***^P<0.001, versus corresponding vehicle; ^†^P<0.05 versus STZ control.
